# Pulmonary Arterial Hypertension Affects the Rat Gut Microbiome

**DOI:** 10.1038/s41598-018-27682-w

**Published:** 2018-06-26

**Authors:** María Callejo, Gema Mondejar-Parreño, Bianca Barreira, José L. Izquierdo-Garcia, Daniel Morales-Cano, Sergio Esquivel-Ruiz, Laura Moreno, Ángel Cogolludo, Juan Duarte, Francisco Perez-Vizcaino

**Affiliations:** 1 Departamento de Farmacología y Toxicología. Facultad de Medicina, Universidad Complutense de Madrid, 28040 Madrid, Spain; 2 Ciber Enfermedades Respiratorias (Ciberes), Madrid, Spain; 3 Instituto de Investigación Sanitaria Gregorio Marañón (IISGM), Madrid, Spain; 4 Fundación Centro Nacional de Investigaciones Cardiovasculares (CNIC), Madrid, Spain; 5 CIC biomaGUNE, Donostia-San Sebastián, Spain; 6 Dept of Pharmacology, Faculty of Pharmacy, University of Granada, Granada, Spain; 7 Ciber Enfermedades Cardiovasculares (CiberCV), Madrid, Spain

## Abstract

We have analysed whether pulmonary arterial hypertension (PAH) alters the rat faecal microbiota. Wistar rats were injected with the VEGF receptor antagonist SU5416 (20 mg/kg s.c.) and followed for 2 weeks kept in hypoxia (10% O_2_, PAH) or injected with vehicle and kept in normoxia (controls). Faecal samples were obtained and microbiome composition was determined by 16S rRNA gene sequencing and bioinformatic analysis. No effect of PAH on the global microbiome was found (α- or β-diversity). However, PAH-exposed rats showed gut dysbiosis as indicated by a taxonomy-based analysis. Specifically, PAH rats had a three-fold increase in Firmicutes-to-Bacteroidetes ratio. Within the Firmicutes phylum, there were no large changes in the relative abundance of the bacterial families in PAH. Among Bacteroidetes, all families were less abundant in PAH. A clear separation was observed between the control and PAH clusters based on short chain fatty acid producing bacterial genera. Moreover, acetate was reduced in the serum of PAH rats. In conclusion, faecal microbiota composition is altered as a result of PAH. This misbalanced bacterial ecosystem might in turn play a pathophysiological role in PAH by altering the immunologic, hormonal and metabolic homeostasis.

## Introduction

Pulmonary arterial hypertension (PAH) is a progressive disease affecting the lung vasculature that is characterized by sustained vasoconstriction, vascular remodelling and *in situ* thrombosis^1^. It evolves into an occlusive arteriopathy with high resistance to blood flow, leading to right heart failure and premature death^2,3^. In recent years, altered immune and inflammatory processes are being considered as pathological hallmarks of the disease^2,3^. In addition, altered metabolism involving a switch to glycolysis, fatty acid oxidation, and production of reactive oxygen species are also being currently recognized in the pathogenesis of PAH^4^.

The human gut is colonized by a huge number of bacteria, archaea, protists, fungi and viruses, forming an ecological community known as the gut microbiota. The gut microbiota communicates with distal organs by producing numerous metabolites that may be absorbed into the systemic circulation and exert biological effects^5^. The microbiota is also responsible for the integrity of the gut barrier function. Low-grade bacterial translocation from the intestines into the circulation with increased plasma bacterial endotoxins (lipopolysaccharides, LPS) may also result from gut barrier dysfunction^6^. In recent years, multiple evidences point to a relationship between the composition of the gut microbiota and an appropriate immunologic, hormonal and metabolic homeostasis^7–9^. The changes in the composition of gut microbiota associated with disease are referred to as dysbiosis. This misbalanced bacterial ecosystem may be therapeutically targeted using probiotics -live strains of selected bacteria- or prebiotics -food components modulating the microbiota^10,11^.

Multiple cardiovascular, metabolic and respiratory diseases such as atherosclerosis, hypertension, heart failure, chronic kidney disease, obesity, type 2 diabetes mellitus and sleep apnoea have been linked to gut dysbiosis^12–15^. This is characterized by a microbial flora that is less diverse and less rich with an increased Firmicutes to Bacteroidetes ratio (F/B)^6,16^. Changes in short chain fatty acids (SCFA) producing bacteria are also characteristic of gut dysbiosis with a decrease in acetate- and butyrate-producing bacteria and an increase in lactate-producing bacterial populations^6,8,16^. Moreover, the meta-analysis of the human studies supports that supplementation with probiotics in disease restores the proper gut microbiota and improves disease biomarkers. For instance, probiotics reduce blood pressure in essential hypertensives^17,18^.

Despite the gut microbiota has been suggested to affect the development of pulmonary vascular disease on a theoretical basis^19^, the microbiome has not been studied so far in the context of preclinical or clinical PAH. We hypothesized that the development of PAH may be associated to changes in the intestinal bacterial composition. Therefore, we investigated the effect of PAH on the faecal microbiome in rats using 16S rRNA metagenomics^20^. We have used a representative animal model of PAH, consisting in the combination of hypoxia plus the VEGF antagonist SU5416^21^. Herein, we report that PAH is associated to gut dysbiosis, namely an increased F/B ratio. This represents the first, but still preliminary, evidence suggesting a possible pathophysiological role of intestinal bacteria in the disease.

## Results

### Hemodynamics and vascular remodelling

**Figure 1:**
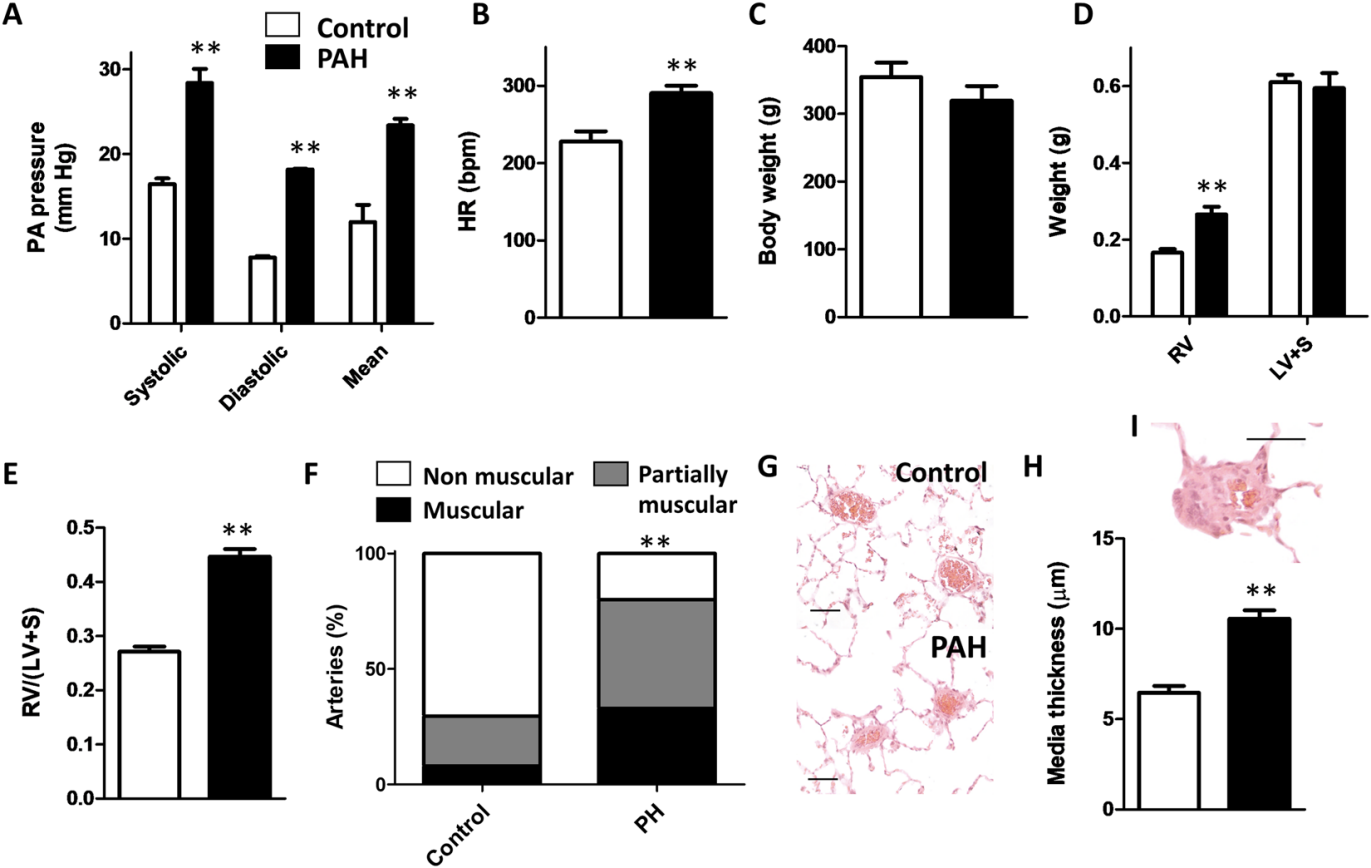
Hemodynamic and histological changes. (**A**) Systolic, diastolic and mean PAP, (**B**) Heart rate, (**C**) Body weight, (**D**) RV and LV+S weight, (**E**) Fulton index [RV/(LV+S)] and (**F**) percentage of arterial muscularization. (**G**) Typical hematoxilin-eosin staining of arterial sections (scale bar 50 µm), (**H**) Medial thickness, (**I**) An early obliterated lesion (scale bar 50 µm). Results are means ± s.e.m. of 4 animals, **p < 0.05 versus control (Students’ t test for panels A–E and Square Chi test for panel F).

SU5416 plus hypoxia for two weeks produced the expected increases in systolic, diastolic and mean pulmonary arterial pressure (PAP) characteristic of PAH (Fig. 1A). This was associated with an increased heart rate, and a trend for reduced body weight (Fig. 1B,C). PAH animals developed a marked right ventricular hypertrophy as shown by the increased RV weight in either absolute values or referred to LV + S (Fig. 1D,E). In addition, animals with PAH showed arterial wall remodelling with an increased muscularization of the small resistance arteries (Fig. 1F,G) with increased wall thickness (Fig. 1H) and occasionally early obliterated lessions were observed (Fig. 1I).

### Bacterial α- and β-diversity

**Figure 2:**
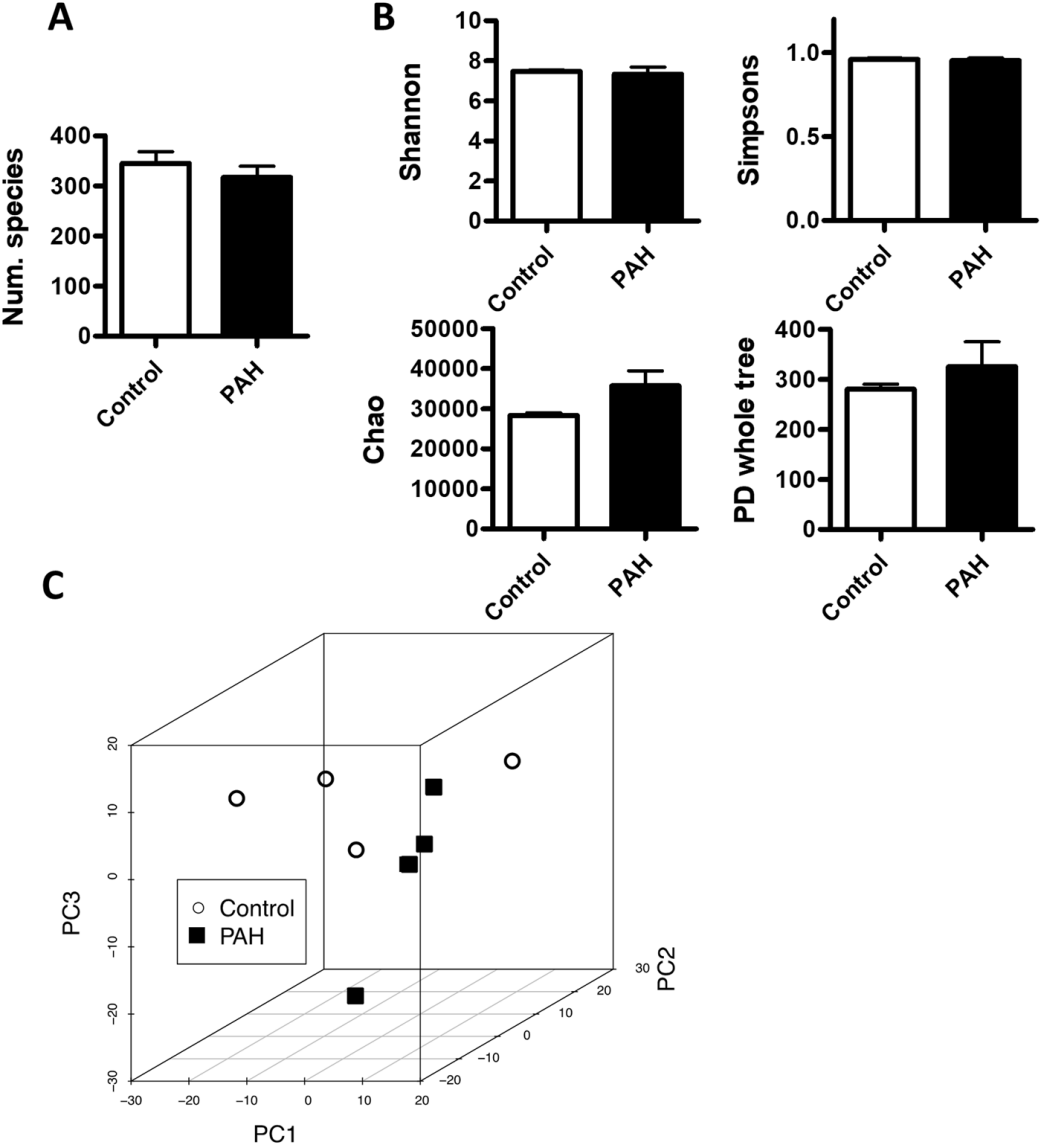
Microbial richness and diversity in PH and Principal Coordinate analysis (PCA). (**A**) Number of species identified. (**B**) α-diversity in rats in control and PAH rats measured by the Shannon, Chao, Simpsons and PD whole tree indexes. Results are means ± s.e.m. of 4 animals. (**C**) Unsupervised PCA were carried out to analyse the differences between control and PAH groups. Each principal component describes most of the variation between samples.

The number of species identified was similar in the control and PAH group (Fig. 2A). Shannon, Chao, Simpson and PD whole tree indexes, which represent both the richness and evenness of its species diversity within each sample, i.e. α-diversity, were also similar in both groups (Fig. 2B). We performed a tridimensional principal component analysis (PCA) of the bacterial community, which measures microorganism diversity between samples, i.e. β-diversity, at the level of the different taxa (phylum, class, order, family, genus and species), in an unsupervised manner. This analysis showed no perfect clustering of the animals into the control and PAH groups; e.g. Fig. 2C shows the analysis at the species level.

### Taxa composition

**Figure 3:**
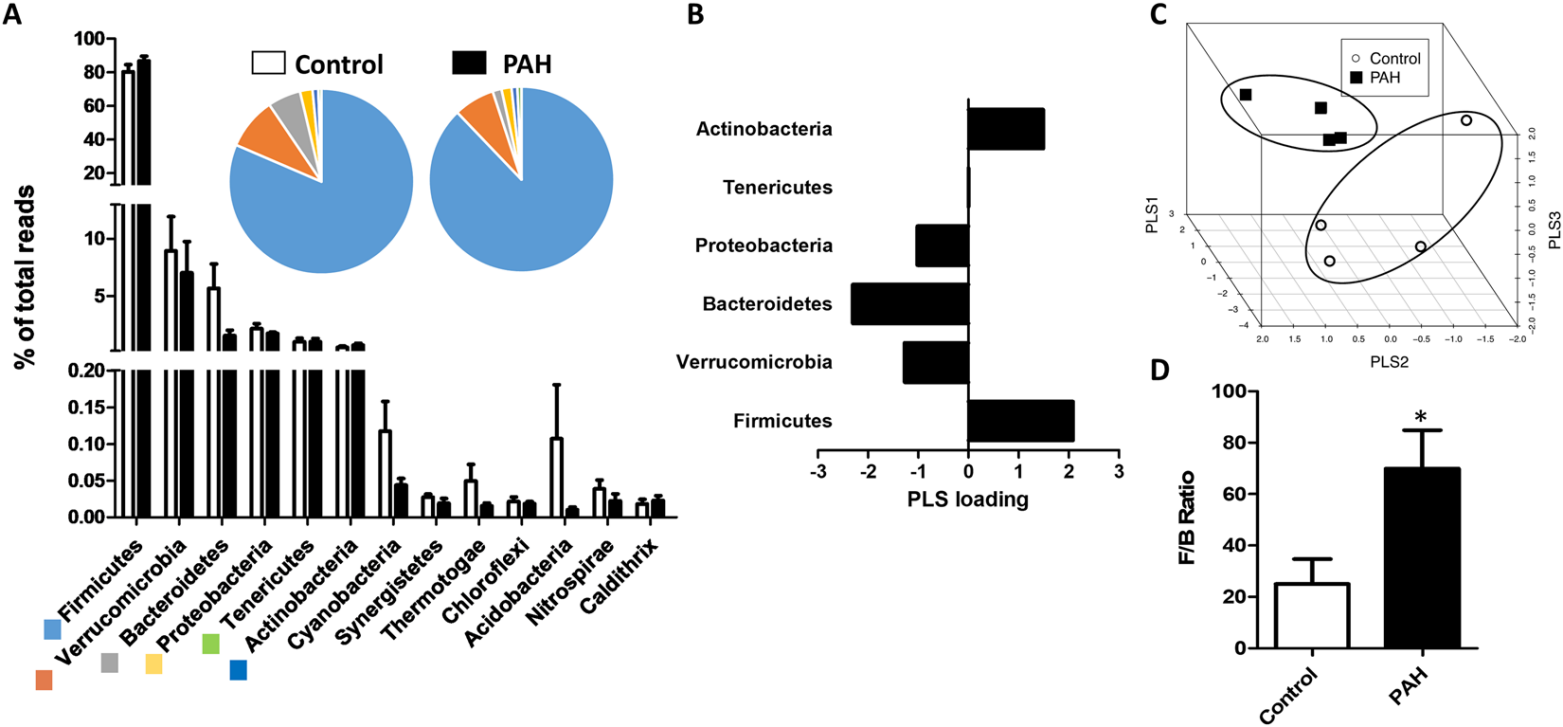
Phyla composition. (**A**) Composition of the most abundant bacterial phyla (>0.01%) expressed as a percent of total bacteria (means ± s.e.m. of 4 animals). The inset shows the pie charts for control and PAH. (**B**) PLS loadings (data shown for phyla representing >0.1% of total bacteria) highlight variable significance to discriminate between PAH and control samples in PLS scores. (**C**) Tridimensional PLS scores plot. (**D**) The Firmicutes to Bacteroidetes ratio (F/B ratio) was calculated as a biomarker of gut dysbiosis (means ± s.e.m., n = 4, *p = 0.04 vs control with student’s t-test).

The analysis of the phyla composition showed that Firmicutes was the most abundant phylum in the rat faeces, followed by Verrucomicrobia, Bacteroidetes, Proteobacteria, Tenericutes and Actinobacteria (Fig. 3A). Each of these taxa represented above 0.5% of total bacteria and altogether accounted for 98.7% and 98.8% of total bacteria in control and PAH groups, respectively. A nearly four-fold relative decrease in the Bacteroidetes phylum (5.7 vs 1.5%) with lower relative changes in the other most abundant phyla was found in the animals treated with hypoxia plus SU5416. Among the less abundant phyla, there was a ≈three-fold decrease in Cyanobacteria-related bacteria and Thermotogae and a ten-fold decrease in Acidobacteria. The Partial Least Square (PLS) loadings which indicate both the magnitude of the change and the statistical probability of the difference are shown in Fig. 3B for phyla representing >0.1% of total bacteria. The most relevant differences were found in the Bacteroidetes phyla. A 3-dimensional scatterplot was generated by PLSR to visualize the differences in composition of the faecal microbial communities (Fig. 3C). A clear separation was observed between the control and PAH clusters. Notably, the calculated F/B, a hallmark of gut dysbiosis, was significantly increased in PAH (Fig. 3C).

**Figure 4:**
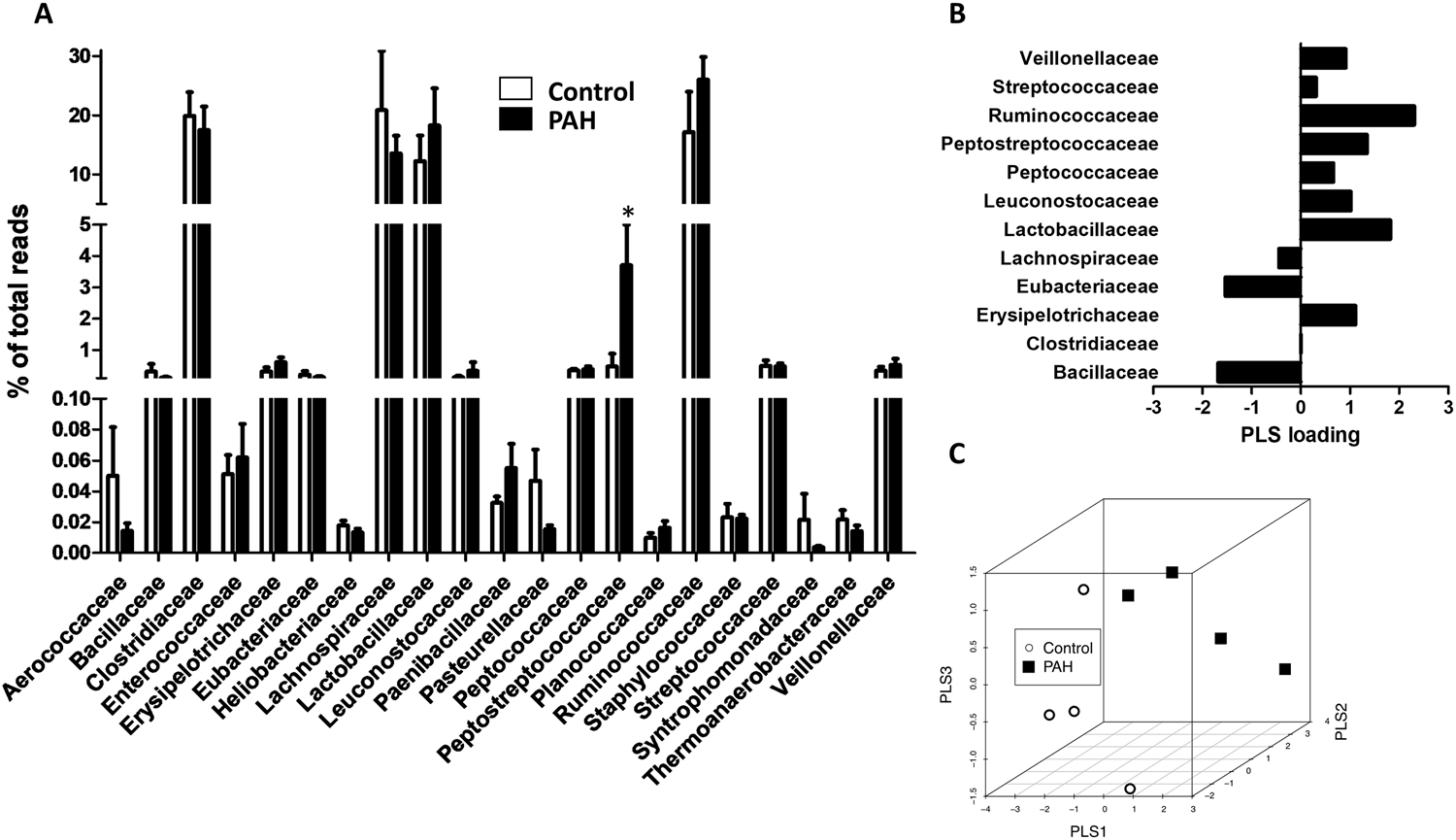
Bacterial families within the Firmicutes phylum. (**A**) Composition of the most abundant bacterial families (>0.01%) expressed as a percent of total bacteria in control and PAH rats (means ± s.e.m. of 4 animals, *p < 0.05 vs control with student’s t-test). (**B**) PLS loadings (data shown for phyla representing >0.1% of total bacteria) highlight variable significance to discriminate between PAH and control samples in PLS scores. (**C**) Tridimensional PLS scores plot.

Given the altered F/B ratio, we analysed which families of bacteria contributed to this imbalance. Regarding the most common families of the Firmicutes phylum, in general, there were no large changes in their relative abundance in the PAH compared to the control group (Fig. 4A,B) with the exception of Peptostreptococcaceae, which suffered a seven-fold increase (0.5 to 3.7%). In the less abundant families, there was also trend for a decrease (≈three-fold) in Aerococcaceae and Pasteurellaceae and a five-fold decrease in Syntrophomonadaceae.

**Figure 5:**
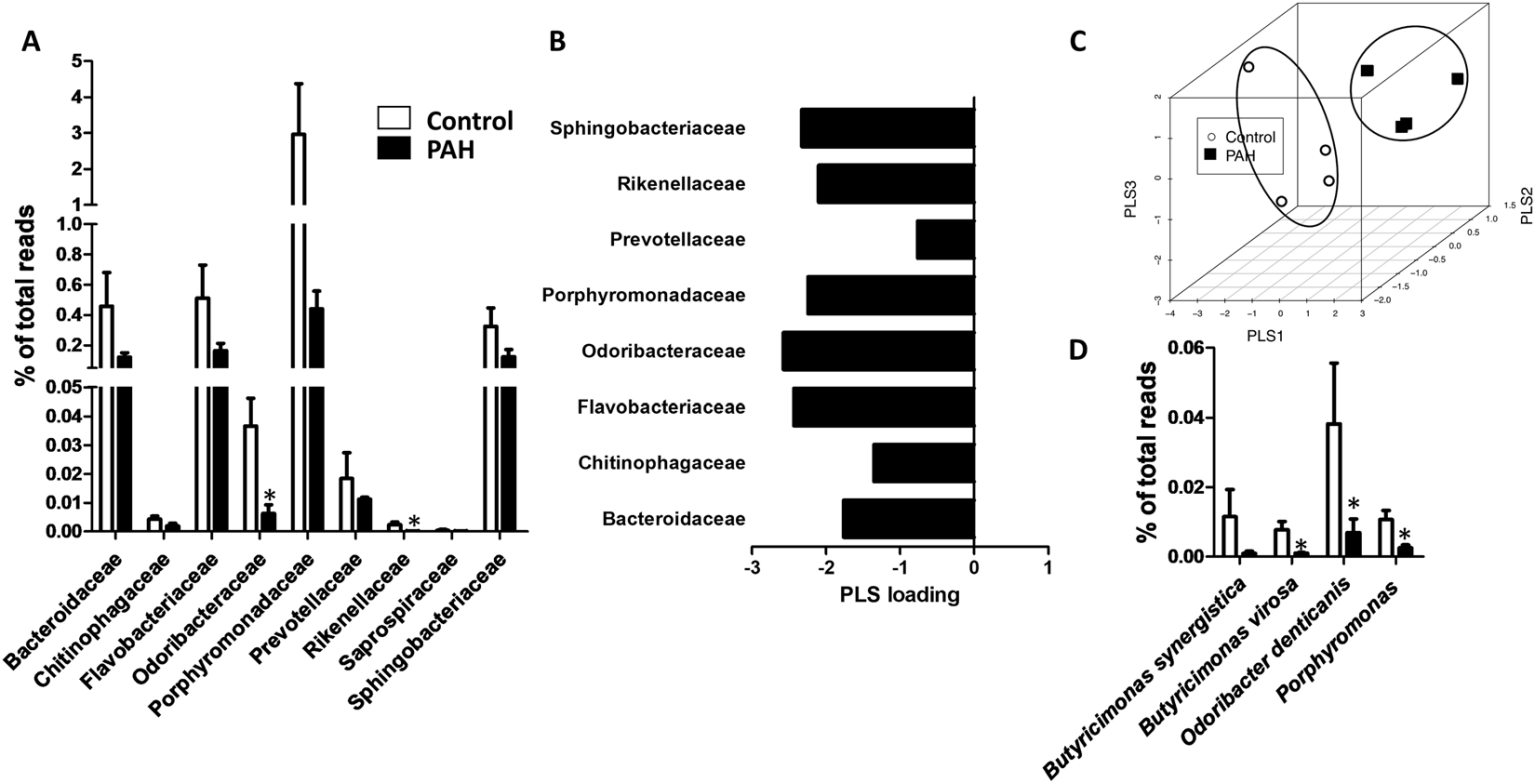
Bacterial families within the Bacteroidetes phylum. (**A**) Composition of the bacterial families expressed as a percent of total bacteria in control and PAH rats (means ± s.e.m. of 4 animals, *p < 0.05 vs control with student’s t-test) (**B**) PLS loadings (data shown for phyla representing >0.1% of total bacteria) highlight variable significance to discriminate between PAH and control samples in PLS scores. (**C**) Tridimensional PLS scores plot. (**D**) Composition of the species within the Odoribacteraceae family and *Porphyromonas* (means ± s.e.m. of 4 animals, *p < 0.05 vs control with student’s t-test).

Among the Bacteroidetes phylum, all families were decreased in PAH (from 2- to 20-fold decrease, Fig. 5A). Figure 5B shows the PLS loadings. The most relevant decreases at the genus level were observed in *Butyricimonas* and *Odoribacter* among Odoribacteraceae and *Porphyromonas* in Porphyromonadaceae (Fig. 5D). PLS analysis of the families within Bacteroidetes (Fig. 5C) clearly separated the control and PAH clusters. *Bifidobacterium*, a commonly considered beneficial genus^22^ that belongs to the Actinobacteria phylum, was not significant different (0.094 ± 0.039% in control and 0.072 ± 0.005% of total reads in PAH).

### SCFA-producing bacteria and SCFA in serum

**Figure 6:**
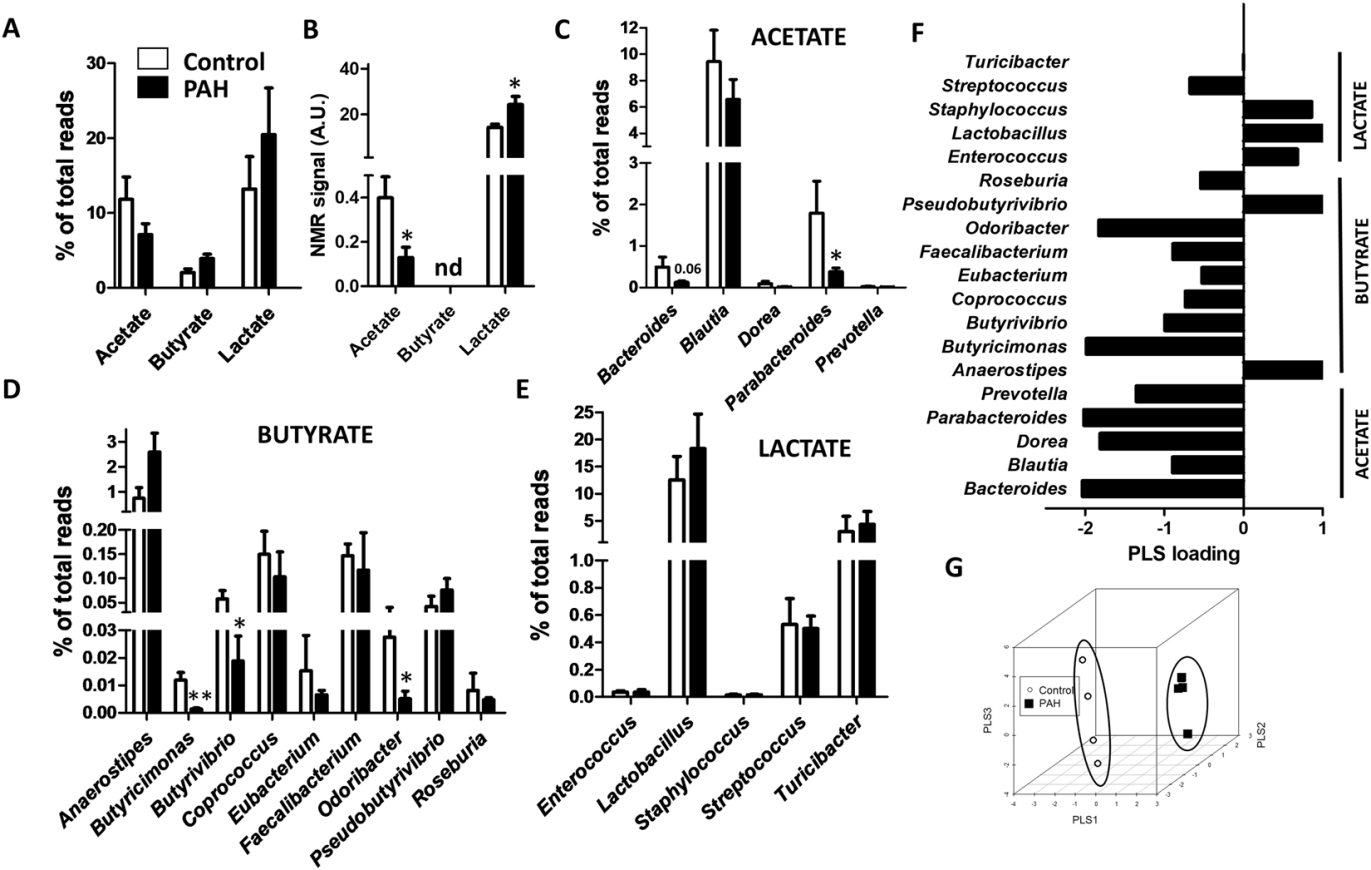
SCFA and SCFA-producing bacteria. (**A**) Composition of the acetate-, butyrate- and lactate-producing bacteria in control and PAH rats. Data is the sum of all SCFA-producing genera expressed as a percent of total bacteria (means ± SEM of 4 animals). (**B**) Acetate, butyrate and lactate in rat serum (AU = arbitrary units, nd = not detected, n = 4, *p < 0.05 vs control student’s t-test). (**C**–**E**) Most abundant acetate-, butyrate- and lactate-producing genera (means ± s.e.m. of 4 animals, *p < 0.05 vs control with student’s t-test). (**F**) PLS loadings (data shown for phyla representing >0.1% of total bacteria) highlight variable significance to discriminate between PAH and control samples in PLS scores. (**G**) Tridimensional PLS scores plot.

We analysed the changes in the relative abundance of SCFA-producing bacteria as another hallmark of gut dysbiosis (Fig. 6A) and the SCFA levels in serum (Fig. 6B).

We found a trend for reduced acetate-producing bacteria that was reproduced for all individual acetate-producing genera and also for most butyrate-producing bacteria (based on PLS loadings as shown in Fig. 6F). A statistically significant reduction was only observed for some acetate-producing and butyrate-producing genera (Student t test, Fig. 6C,D). However, an overall trend for increased butyrate-producing bacteria was driven by the changes in the most abundant genus Anaerostipes (Fig. 6D). Lactate-producing bacteria were essentially unchanged (Fig. 6A,E and F). We also analysed the serum levels of SCFA from the NMR spectra (Fig. 6B). Acetate was significantly decreased in serum while butyrate levels were not detected in the NMR spectra. In contrast, there was increased serum levels of lactate (P < 0.01) in PAH vs control animals (Fig. 6B). The PLS analysis clearly separated the control and PAH clusters based on SCFA-producing bacteria (Fig. 6G).

## Discussion

The role of gut dysbiosis in the pathogenesis of many diseases, including diabetes mellitus, obesity, cancer, psychiatric, respiratory and cardiovascular disorders is rapidly emerging. In this study, we present the first evidence of changes in the microbiota in a small sample of rats during the early phases of PAH. Notably, we found an increased F/B that is considered the hallmark of gut dysbiosis, in a rat model of PAH. We also found some specific changes in several taxa, which reproduce the changes previously observed associated to other cardiovascular and metabolic diseases.

We have used the animal model of PAH of hypoxia plus SU5416, which best conforms to human PAH^21^. At two weeks, it develops a clear increase in PAP at the threshold values for clinical diagnosis and strong right ventricular hypertrophy and arterial remodelling. We deliberately chose this time to analyse the early changes in the microbiota, which might play a pathophysiological role in the development of the pathology rather than being a consequence of the long-term disease. Longer exposure leads to further disease worsening. For instance, at baseline and after the second and the third week of treatment, mPAP increases from ≈13 to 24 (present data) and 45 mm Hg (authors unpublished data), respectively. This time-course is similar to the one shown in the original report of the model by Taraseviciene-Stewart *et al*.^23^ and consistent with RV pressure values of ≈30, 60 and 90 mm Hg, respectively, in Oka *et al*.^24^. Likewise, the Fulton indexes as a measure of RV hypertrophy were ≈0.25, 0.45 and 0.65, respectively, in our hands and ≈0.3, 0.5 and 0.7, respectively, in Oka *et al*.^24^.

The Shannon, Simpsons, Chao and PD whole tree indexes and the PCA plot showed no apparent change in α- and β-diversity in controls and PAH rats. This indicates that there is no global differences in the microbiota, most taxa were unchanged. In contrast, other diseases have found reduced richness and diversity^6,16^. However, the most important and recognized biomarker of dysbiosis, the F/B, was significantly increased in PAH. This ratio has been reported to be modified in multiple pathological conditions in both human and animal models. In systemic hypertension increased F/B has been found in animal models of disease, including spontaneously hypertensive rats, deoxycorticosterone-salt- and angiotensin II-induced hypertension, as well as in essential hypertensive patients^16,25^.

We also evaluated which subtaxa contributed to the alteration of Firmicutes and Bacteroidetes. Notably, all families from the Bacteroidetes phylum were decreased (from 2 to 10-fold decrease). Odoribacteraceae may be of special interest because several species within this family belonging to the genera *Odoribacter* and *Butyricimonas* have been reported to be depleted in overweight and obese pregnant women with high blood pressure, in sedentary mice, liver injury and multiple sclerosis^26–28^. In contrast to the present report, *Odoribacter* was found to be increased in mice with intermittent hypoxia^29^. Among Firmicutes, we found minor absolute changes, with an overall trend for an increase in bacterial reads. Peptostreptococcaceae, which suffered the largest absolute increase, is a family of Gram-positive bacteria that is over-represented in the guts of patients and mice with colorectal and oral cancer^30,31^.

Dietary fibre is fermented in the colon by commensal bacteria, leading to the release of the SCFAs, acetate, butyrate, and lactate, which may be absorbed into the circulation and interact with G protein-coupled olfactory receptors in the gut epithelium and immune cells^32^. Besides the changes in taxonomic categories, gut dysbiosis associated to cardiovascular disease is characterized by a decrease in acetate- and butyrate-producing bacteria and an increase in lactate-producing bacterial populations^6,8,16^. We found no significant changes in the sum of butyrate- or lactate-producing bacteria but a trend for reduced acetate-producing bacteria was found in PAH. A clear separation was observed between the control and PAH clusters based on short chain fatty acid producing bacterial genera. We also analyzed the levels of the three SCFA in serum of the PAH rats and controls by quantifying the NMR spectra. We found that acetate was reduced in the serum of PAH rats. This change parallels the observed differences in acetate-producing genera. It is therefore tempting to speculate that the observed serum changes are secondary to the different bacterial composition. However, our experiments cannot rule out that the observed changes in serum SCFA are generated by the host metabolism. In fact, lactate was increased in the serum of PAH rats, which is expected as a result of the hypoxic environment in the host cells. Interestingly, acetate supplementation or an intervention with fibre to restore acetate production in mice with mineralocorticoid-dependent hypertension significantly reduced systolic and diastolic blood pressures, cardiac fibrosis, and left ventricular hypertrophy^25^. These protective effects seem to be related to the regulation of key pathways and genes involved in cardiovascular health, including the transcription factor Egr1, a master regulator of cardiovascular disease^33,34^ which has also been reported to play a role in PAH^35^.

Interestingly, there is certain parallelism between factors affected by gut dysbiosis and those involved in the pathophysiology of PAH. First, it is now well established that Th17 cell development in the gut is specifically impacted by commensal bacteria^36^. Th17 cell expansion originated in the intestine is associated with gut dysbiosis (higher F/B ratio) in several pathologies such as multiple sclerosis^37^ and lupus erythematosus^38^. A characteristic increase in peripheral Th17 cells and the Th17-produced cytokine IL-17 and a decrease in Treg cells is common to all forms of PAH and contributes to the development and the progression of the disease^39,40^. Second, gut microbiota is also a key regulator of Tph1 transcription (the gene encoding for the rate limiting enzyme in serotonin synthesis) in enterochromaffin cells which supplies the platelets of serotonin^41^. On the other hand, clinical and experimental PAH is associated with up-regulation of Tph1 gene transcripts as well as a rise in platelet-rich serotonin^21,42^. Third, gut dysbiosis leads to low grade commensal bacterial translocation^6,7^ with increased plasma bacterial LPS, the main ligand for toll-like receptor 4 (TLR4). This innate immune receptor has been reported to play a key role in the pathogenesis of pulmonary hypertension^43^. Therefore, there might be a pathophysiological link between gut dysbiosis and PAH that involves the upregulation of Egr1, Th17 polarization, elevation of plasma serotonin and TLR4 activation.

The mechanism of how PAH induces gut dysbiosis remains to be determined. It has been reported that the sympathetic nervous system, via beta-adrenoceptor activation, in the gut compromises its barrier function, and it is capable of altering the microbiota^44,45^. Interestingly, PAH patients and animal models (including chronic hypoxia plus SU5416-induced PAH) have high sympathetic activity and circulating catecholamine levels^46,47^, which is strongly related to mortality^48^. Therefore, it seems reasonable to tentatively propose sympathetic overstimulation as a mechanism for PAH-induced gut dysbiosis. If this is the case, neurohumoral activation might exert deleterious effects in PAH not only by adrenergic receptor stimulation on the heart and pulmonary vessels but also on the splachnic circulation. However, we cannot rule out that the effects of hypoxia and SU5416 may not be limited to the pulmonary vasculature and could impact directly on other tissues, including the gut epithelium and/or the mesenteric vasculature, leading to gut dysbiosis. Moreover, we cannot exclude that the gut is a primary target of hypoxia and/or SU5416 and that the subsequent changes in the microbiota secondarily trigger or potentiate PAH. In addition, our experiments do not clarify whether the changes in the microbiota are induced by hypoxia, SU5416 or the combination of both. SU5416 itself induces mild pulmonary hypertension^23^ and lung cell apoptosis and emphysema^49^. A possible strategy to address these issues could be to treat animals with SU5416 by inhalation to minimize the direct systemic effects. However, to our knowledge, there are no reports using this administration route for this drug.

In conclusion, the present study is the first one showing that PAH affects the gut microbiota. Further research is required to determine whether dysbiosis plays a pathophysiological role in the development of PAH or if it is just an epiphenomenon. If the former is true, a new therapeutic window will be opened in PAH. Several therapeutic strategies can be used to restore the microbiota in disease^10,11,17,25^, including specific bacterial strains (probiotics), fibre and dietary polyphenols (i.e. prebiotics), faecal transplantation, antibiotics, beta-adrenergic antagonists^45^ or to replace the deficit in specific SCFAs (e.g. acetate)^25^.

## Material and Methods

### Animals

Pathogen-free male Wistar rats (300 g, 11–12 weeks of age) were obtained from Envigo (Barcelona, Spain). All experimental procedures utilizing animals were carried out according to the Spanish Royal Decree 1201/2005 and 53/2013 on the Care and Use of Laboratory Animals and approved by the institutional Ethical Committees of the Universidad Complutense de Madrid (Madrid, Spain) and the regional Committee for Laboratory Animals Welfare (Comunidad de Madrid, Ref. number PROEXO-301/16).

### Model of PAH

PAH was induced in rats by a single subcutaneous injection of SU5416 (20 mg/kg; Tocris, UK) and then maintained in hypoxia for two weeks^21^. Hypoxic animals (n = 4) breathed a gas mixture (N_2_ and room air) in a semi-closed chamber where oxygen was continuously monitored by an oxygen sensor (DrDAQ, PicoTechnology, UK) to maintain 10% O_2_. Control animals (n = 4) were exposed to rom air (21% O_2_, normoxia) in another chamber. CO_2_ and water vapour produced by the animals were captured with soda lime and silica gel, respectively. Animals were fed normal rat chow.

### Hemodynamic measurements

At the end of two weeks, rats were anesthetized (80 mg/kg ketamine and 8 mg/kg xylacine i.p.), tracheostomyzed and ventilated with room air (tidal volume 9 mL/kg, 60 breaths/min, and a positive end-expiratory pressure of 2 cm H_2_O, Nemi Scientific Inc, Medway, USA). After sternotomy, a catheter was placed in the pulmonary artery (PA) through the right ventricle for systolic, diastolic and mean PA pressure (sPAP, dPAP and mPAP) recording^50^. It should be noted that open-chest measurements in anaesthetized animals underestimate real PAP. At the end of the experiment, the right ventricle (RV) and the left ventricle plus the septum (LV + S) were dissected and weighed.

### Lung histology

The left lung was inflated *in situ* with formol saline through the left bronchus and embedded in paraffin. Lung sections were stained with haematoxylin and eosin and examined by light microscopy, and elastin was visualized by its green auto-fluorescence. Small arteries (25–100 mm outer diameter) were analysed in a blinded fashion and categorized as muscular, partially muscular or non-muscular as previously described^50^.

### DNA Extraction, 16S rRNA Gene Amplification, Bioinformatics

For the analysis of the bacterial population present in the gut, faecal samples were collected from four individual animals at the end (day 14) of the experimental period. Bacterial genomic DNA was extracted from faecal samples using G-spin columns (INTRON Biotechnology) starting from 30 mg of samples resuspended in PBS and treated with proteinase K and RNAses. DNA concentration was determined in the samples using Quant-IT PicoGreen reagent (Thermo Fischer) and DNA samples (about 3 ng) were used to amplify the V3-V4 region of 16S rRNA gene^51^. PCR products (approx. 450 pb) included extension tails which allowed sample barcoding and the addition of specific Illumina sequences in a second low-cycle number PCR. Individual amplicon libraries were analysed using a Bioanalyzer 2100 (Agilent) and a pool of samples was made in equimolar amounts. The pool was further cleaned, quantified and the concentration estimated by real time PCR (Kapa Biosystems). Finally, DNA samples were sequenced on an Illumina MiSeq instrument with 2 × 300 paired-end read sequencing at the Unidad de Genómica (Parque Científico de Madrid). Negative controls included from the beginning of the procedure were completely negative and therefore not included in the sequencing run. We did not carry positive controls in our experiments since these primers have been extensively used^51^. The two-step PCR amplification that we have used^52^ allows the successful recovery of mock community species. Our approach to increase diversity included: (a) running different projects in the same run so that proximal clusters can easily start with different sequences; (b) increasing the percentage of PhiX174 DNA, to further increase diversity with an equilibrated shotGun DNA; and (c) diluting cluster density to suboptimal concentration for Miseq v3 runs. DNA reads were quality filtered according to MiSeq standard parameters (Illumina) resulting in a final output of around 150 K reads on average per rat (range: 90–220 K). Operational taxonomic units (OTUs) were assigned using the 16S-metagenomics workflow (1.0.1) associated to the Base Space Hub (Illumina). Classification was based on an Illumina-curated version of the GreenGenes taxonomic database which implements the Ribosomal Database Project (RDP) Classifier^53^. The Taxonomy Database (National Center for Biotechnology Information) was used for classification and nomenclature. Bacteria were classified based on the SCFA end product as previously described^54,55^.

### Serum SCFA measurements

Serum samples (40 µL) were examined by 500 MHz High-Resolution Magic Angle Spinning Nuclear Magnetic Resonance Bruker AMX500 spectrometer at CIC Biomagune (Donostia, Spain). Samples were placed into a 50-µl zirconium oxide rotor using a rinsed cylindrical insert, together with 15 µl 0.1 mM solution Trimethylsilyl propanoic acid (TSP) in deuterium water (D2O). Standard solvent-suppressed spectra were acquired using a sequence based on the first increment of the nuclear Overhauser effect spectroscopy (NOESY) pulse sequence. A number of bidimensional homonuclear and heteronuclear experiments such as standard gradient-enhanced correlation spectroscopy (COSY), ^1^H–^1^H total correlated spectroscopy (TOCSY), and gradient-selected heteronuclear single quantum correlation (HSQC) protocols were performed to carry out metabolites assignments. Spectral processing was performed using the “Metabonomic” R package^56^. 1H NMR spectra were referenced to the TSP signal at 0 ppm chemical shift and normalized to total sum of the spectral regions. Two-dimensional spectral processing and editing was performed using MestRenova v. 11.0.3 (Mestrelab Research S.L., Santiago de Compostela, Spain).

### Statistical analysis

The Shannon, Chao, Simpsons and PD whole tree indexes were calculated to analyse α-diversity using QIIME. Reads in each OUT were normalized to total reads in each sample. Only taxa with a percentage of reads >0.001% were used for the analysis. Data are expressed as means ± s.e.m. Statistical comparisons were performed using two-tailed unpaired *t* tests at α < 0.05 where appropriate. Unsupervised classification studies with Principal Components Analysis (PCA)^57^ were carried out to analyse the differences between groups for each taxonomic level. Partial Least Square (PLS) analysis was also applied to these data to identify significant differences between groups. PLS analysis^58^ is a commonly used supervised multivariate method for analysing high-dimensional data where PLS loadings highlight the most significant variables from the total pool. The PLS components are composed of so-called scores and loadings. PLS loadings contain information about the variables in the dataset highlighting the most significant variables from the total pool. PLS scores hold information on samples in the dataset highlighting the differences between groups. PLS analyses were performed with the Metabonomic package (rel.3.3.1)^56^ using the algorithm proposed by Ding and Gentleman^59^. Three PLS components were chosen to build the model based on the percentage of variance explained, the R2, and the mean squared error of cross-validation graphics.

### Data availability

All data of the present study are available on request.

## References

[1] Galie N (2016). 2015 ESC/ERS Guidelines for the diagnosis and treatment of pulmonary hypertension: The Joint Task Force for the Diagnosis and Treatment of Pulmonary Hypertension of the European Society of Cardiology (ESC) and the European Respiratory Society (ERS): Endorsed by: Association for European Paediatric and Congenital Cardiology (AEPC), International Society for Heart and Lung Transplantation (ISHLT). Eur. Heart J..

[2] Rabinovitch M, Guignabert C, Humbert M, Nicolls MR (2014). Inflammation and immunity in the pathogenesis of pulmonary arterial hypertension. Circ. Res..

[3] Schermuly RT, Ghofrani HA, Wilkins MR, Grimminger F (2011). Mechanisms of disease: pulmonary arterial hypertension. Nat. Rev. Cardiol..

[4] D’Alessandro A (2017). Hallmarks of Pulmonary Hypertension: Mesenchymal and Inflammatory Cell Metabolic Reprogramming. Antioxid. Redox Signal..

[5] Fu ZD, Cui JY (2017). Remote Sensing between Liver and Intestine: Importance of Microbial Metabolites. Curr. Pharmacol. Rep..

[6] Marques FZ, Mackay CR, Kaye DM (2017). Beyond gut feelings: how the gut microbiota regulates blood pressure. Nat. Rev. Cardiol..

[7] Kamada N, Seo SU, Chen GY, Nunez G (2013). Role of the gut microbiota in immunity and inflammatory disease. Nat. Rev. Immunol..

[8] Maslowski KM (2009). Regulation of inflammatory responses by gut microbiota and chemoattractant receptor GPR43. Nature.

[9] McDermott AJ, Huffnagle GB (2014). The microbiome and regulation of mucosal immunity. Immunology.

[10] Markowiak, P. & Slizewska, K. Effects of Probiotics, Prebiotics, and Synbiotics on Human Health. *Nutrients* **9**, E1021, 1010.3390/nu9091021 (2017). 10.3390/nu9091021 PMC5622781 28914794

[11] Kristensen NB (2016). Alterations in fecal microbiota composition by probiotic supplementation in healthy adults: a systematic review of randomized controlled trials. Genome Med..

[12] Tang WH, Kitai T, Hazen SL (2017). Gut Microbiota in Cardiovascular Health and Disease. Circ. Res..

[13] Tang WH, Hazen SL (2014). The contributory role of gut microbiota in cardiovascular disease. J. Clin. Invest..

[14] Moreno-Indias I (2015). Intermittent hypoxia alters gut microbiota diversity in a mouse model of sleep apnoea. Eur. Respir. J..

[15] Shukla SD, Budden KF, Neal R, Hansbro PM (2017). Microbiome effects on immunity, health and disease in the lung. Clin. Transl. Immunology.

[16] Yang T (2015). Gut dysbiosis is linked to hypertension. Hypertension.

[17] Robles-Vera I (2017). Antihypertensive Effects of Probiotics. Curr. Hypertens. Rep..

[18] Gomez-Guzman M (2015). Antihypertensive effects of probiotics Lactobacillus strains in spontaneously hypertensive rats. Mol. Nutr. Food Res..

[19] Kinlay S, Michel T, Leopold JA (2016). The Future of Vascular Biology and Medicine. Circulation.

[20] Jovel, J. *et al*. Characterization of the Gut Microbiome Using 16S or Shotgun Metagenomics. *Front. Microbiol*. **7**, 459, 410.3389/fmicb.2016.00459 (2016). 10.3389/fmicb.2016.00459 PMC4837688 27148170

[21] Ryan JJ, Marsboom G, Archer SL (2013). Rodent models of group 1 pulmonary hypertension. Handb. Exp. Pharmacol..

[22] Fukuda S (2011). Bifidobacteria can protect from enteropathogenic infection through production of acetate. Nature.

[23] Taraseviciene-Stewart L (2001). Inhibition of the VEGF receptor 2 combined with chronic hypoxia causes cell death-dependent pulmonary endothelial cell proliferation and severe pulmonary hypertension. FASEB J.

[24] Oka M (2007). Rho kinase-mediated vasoconstriction is important in severe occlusive pulmonary arterial hypertension in rats. Circ Res.

[25] Marques FZ (2017). High-Fiber Diet and Acetate Supplementation Change the Gut Microbiota and Prevent the Development of Hypertension and Heart Failure in Hypertensive Mice. Circulation.

[26] Fang D (2017). Bifidobacterium pseudocatenulatum LI09 and Bifidobacterium catenulatum LI10 attenuate D-galactosamine-induced liver injury by modifying the gut microbiota. Sci. Rep..

[27] Gomez-Arango LF (2016). Increased Systolic and Diastolic Blood Pressure Is Associated With Altered Gut Microbiota Composition and Butyrate Production in Early Pregnancy. Hypertension.

[28] Liu, Z. H. *et al*. Moderate-Intensity Exercise Affects Gut Microbiome Composition and Influences Cardiac Function in Myocardial Infarction Mice. *Front. Microbiol*. **8**, 1687, 1610.3389/fmicb.2017.01687 (2017). 10.3389/fmicb.2017.01687 PMC5585143 28919891

[29] Moreno-Indias I (2016). Normoxic Recovery Mimicking Treatment of Sleep Apnea Does Not Reverse Intermittent Hypoxia-Induced Bacterial Dysbiosis and Low-Grade Endotoxemia in Mice. Sleep.

[30] Ahn J (2013). Human Gut Microbiome and Risk for Colorectal Cancer. J. Natl. Cancer. Inst..

[31] Schulz MD (2014). High-fat-diet-mediated dysbiosis promotes intestinal carcinogenesis independently of obesity. Nature.

[32] Sivaprakasam S, Prasad PD, Singh N (2016). Benefits of short-chain fatty acids and their receptors in inflammation and carcinogenesis. Pharmacol. Therapeut..

[33] Khachigian LM (2006). Early growth response-1 in cardiovascular pathobiology. Circ. Res..

[34] Ho LC (2016). Egr-1 deficiency protects from renal inflammation and fibrosis. J. Mol. Med. (Berl).

[35] Nozik-Grayck E (2008). Lung EC-SOD overexpression attenuates hypoxic induction of Egr-1 and chronic hypoxic pulmonary vascular remodeling. Am. J. Physiol. Lung Cell Mol. Physiol..

[36] Lee YK, Mazmanian SK (2010). Has the Microbiota Played a Critical Role in the Evolution of the Adaptive Immune System?. Science.

[37] Cosorich I (2017). High frequency of intestinal TH17 cells correlates with microbiota alterations and disease activity in multiple sclerosis. Sci. Adv..

[38] Lopez P (2016). Th17 responses and natural IgM antibodies are related to gut microbiota composition in systemic lupus erythematosus patients. Sci. Rep..

[39] Huertas A (2016). Regulatory T Cell Dysfunction in Idiopathic, Heritable and Connective Tissue-Associated Pulmonary Arterial Hypertension. Chest.

[40] Gaowa, S. *et al*. Effect of Th17 and Treg Axis Disorder on Outcomes of Pulmonary Arterial Hypertension in Connective Tissue Diseases. *Mediators Inflamm*. **2014**, 247372, doi: 247310.241155/242014/247372 (2014). 10.1155/2014/247372 PMC4158110 25214713

[41] Yano JM (2015). Indigenous bacteria from the gut microbiota regulate host serotonin biosynthesis. Cell.

[42] MacLean MR, Dempsie Y (2010). The Serotonin Hypothesis of Pulmonary Hypertension Revisited. Adv. Exp. Med. Biol..

[43] Bauer EM (2014). Genetic deletion of toll-like receptor 4 on platelets attenuates experimental pulmonary hypertension. Circ. Res..

[44] Sun Y, Fihn BM, Sjovall H, Jodal M (2004). Enteric neurones modulate the colonic permeability response to luminal bile acids in rat colon *in vivo*. Gut.

[45] Stanley D (2016). Translocation and dissemination of commensal bacteria in post-stroke infection. Nat. Med..

[46] Usui S (2006). Upregulated neurohumoral factors are associated with left ventricular remodeling and poor prognosis in rats with monocrotaline-induced pulmonary arterial hypertension. Circ. J..

[47] Piao L (2012). GRK2-Mediated Inhibition of Adrenergic and Dopaminergic Signaling in Right Ventricular Hypertrophy Therapeutic Implications in Pulmonary Hypertension. Circulation.

[48] de Man FS, Handoko ML, Guignabert C, Bogaard HJ, Vonk-Noordegraaf A (2013). Neurohormonal Axis in Patients with Pulmonary Arterial Hypertension Friend or Foe?. Am. J. Respir. Crit Care Med..

[49] Kasahara Y (2000). Inhibition of VEGF receptors causes lung cell apoptosis and emphysema. J Clin Invest.

[50] Morales-Cano D (2014). The flavonoid quercetin reverses pulmonary hypertension in rats. PLoS One.

[51] Caporaso JG (2011). Global patterns of 16S rRNA diversity at a depth of millions of sequences per sample. Proc. Natl. Acad. Sci USA.

[52] Wu L (2015). Phasing amplicon sequencing on Illumina Miseq for robust environmental microbial community analysis. BMC Microbiol.

[53] Wang Q, Garrity GM, Tiedje JM, Cole JR (2007). Naive Bayesian classifier for rapid assignment of rRNA sequences into the new bacterial taxonomy. App. Environ. Microb..

[54] Antharam VC (2013). Intestinal dysbiosis and depletion of butyrogenic bacteria in Clostridium difficile infection and nosocomial diarrhea. J. Clin. Microbiol..

[55] Vital, M., Howe, A. C. & Tiedje, J. M. Revealing the bacterial butyrate synthesis pathways by analyzing (meta)genomic data. *MBio* **5**, e00889, doi: 00810.01128/mBio.00889-00814 (2014). 10.1128/mBio.00889-14 PMC3994512 24757212

[56] Izquierdo-Garcia JL (2009). A novel R-package graphic user interface for the analysis of metabonomic profiles. BMC Bioinformatics.

[57] Hotelling H (1933). Analysis of a complex of statistical variables into principal components. J. Educ. Psychol..

[58] Kramer, R. *Chemometric techniques for quantitative analysis*. (Marcel Dekker 1998).

[59] Ding BY, Gentleman R (2005). Classification using generalized partial least squares. J. Comput. Graph. Stat..

